# Adaptive Evolution of Synthetic Cooperating Communities Improves Growth Performance

**DOI:** 10.1371/journal.pone.0108297

**Published:** 2014-10-09

**Authors:** Xiaolin Zhang, Jennifer L. Reed

**Affiliations:** 1 Cellular and Molecular Biology, University of Wisconsin-Madison, Madison, Wisconsin, United States of America; 2 Department of Chemical and Biological Engineering, University of Wisconsin-Madison, Madison, Wisconsin, United States of America; Virginia Commonwealth University, United States of America

## Abstract

Symbiotic interactions between organisms are important for human health and biotechnological applications. Microbial mutualism is a widespread phenomenon and is important in maintaining natural microbial communities. Although cooperative interactions are prevalent in nature, little is known about the processes that allow their initial establishment, govern population dynamics and affect evolutionary processes. To investigate cooperative interactions between bacteria, we constructed, characterized, and adaptively evolved a synthetic community comprised of leucine and lysine *Escherichia coli* auxotrophs. The co-culture can grow in glucose minimal medium only if the two auxotrophs exchange essential metabolites — lysine and leucine (or its precursors). Our experiments showed that a viable co-culture using these two auxotrophs could be established and adaptively evolved to increase growth rates (by ∼3 fold) and optical densities. While independently evolved co-cultures achieved similar improvements in growth, they took different evolutionary trajectories leading to different community compositions. Experiments with individual isolates from these evolved co-cultures showed that changes in both the leucine and lysine auxotrophs improved growth of the co-culture. Interestingly, while evolved isolates increased growth of co-cultures, they exhibited decreased growth in mono-culture (in the presence of leucine or lysine). A genome-scale metabolic model of the co-culture was also constructed and used to investigate the effects of amino acid (leucine or lysine) release and uptake rates on growth and composition of the co-culture. When the metabolic model was constrained by the estimated leucine and lysine release rates, the model predictions agreed well with experimental growth rates and composition measurements. While this study and others have focused on cooperative interactions amongst community members, the adaptive evolution of communities with other types of interactions (e.g., commensalism, ammensalism or parasitism) would also be of interest.

## Introduction

Microbes are affected by their physical and chemical environment, and they naturally encounter other species that can also influence their behaviors. Symbiotic interactions between microbes and higher organisms can lead to stable interactions and microbial communities. Mutualism is one type of symbiotic interaction, where both species benefit from the interaction. The existence of cooperation between members of a community appears to violate evolutionary theory that natural selection favors selfish behaviors, and therefore different theories have been proposed to explain how cooperation arises and evolves [1]–[5]. While symbiotic interactions are important, most of our knowledge of bacterial metabolism has been gathered from studies of individual strains in pure cultures. However, more than 99 percent of microbes cannot be cultured in mono-culture, since their growth depends on the presence of other species [6]. Additionally, the phenotypes of cultivatable strains may drastically change when grown in a mixed community as compared to mono-culture [7], [8]. Therefore, studies are needed on how bacteria interact in mixed cultures.

In the last decade, experimental efforts have been made to build and study controlled multi-species/strain systems [9]–[12]. Hosada et al. used amino acid *Escherichia coli* auxotrophs to investigate requirements for nascent cooperation, including how initial cell concentrations affect co-culture dynamics [11]. Wintermute and Silver screened 1,035 combinations of *E. coli* auxotrophs to identify pairs of strains that could grow in co-culture and estimated cooperation levels and costs associated with cooperation between strains grown in co-culture [10]. Kerner et al. created a tunable system using tyrosine and tryptophan *E. coli* auxotrophs containing inducible genetic circuits that control production of tyrosine and tryptophan, and thus growth rates and strain ratios [9]. Recently, Pande et al. studied co-cultures of cross-feeding *E. coli* mutants which consumed (due to an amino acid auxotrophy) and produced amino acids. Surprisingly, they showed that most co-cultures with cross-feeders had faster growth rates than the wild-type strains and were stable in the presence of non-cooperators [12].

While these studies investigated initial stages of cooperation in co-culture, other studies have also investigated how adaptive evolution alters community behaviors. Harcombe used co-cultures of a methionine *E. coli* auxotroph and a methionine secreting *Salmonella typhimurium* to select for improved methionine secretion [13]. Harcombe showed that adaptive evolution of co-cultures, made up of three strains, selected for cooperators (methionine secreting *S. typhimurium*) over non-cooperators (wild-type *S. typhimurium*) and that loss of spatial structure (by using flasks rather than agar plates) led to a loss of cooperators over time. Hillesland et al. adaptively evolved co-cultures of a sulfate reducing bacterium and a methanogenic archae and found growth rates and biomass yields improved significantly (by 80% and 30%, respectively). When evolved populations were co-cultured with their ancestral partner, antagonistic interactions were found between the two evolved populations [14].

Mathematical models have also been used to explore natural and synthetic co-cultures of microbes. Using parameters measured in co-cultures of two auxotrophic yeast strains, Shou et al. delineated requirements for initial cell densities and cell numbers needed to achieve an initial viable co-culture [15]. Bull and Harcombe used a model of two cross-feeding species to show how population dynamics affected the fitness of the microbial community [16]. Constraint-based metabolic models have also been used to study natural and synthetic microbial communities. These models have be used to identify strains capable of cooperating, predict intra- and extra-cellular flux distributions in co-cultures [17], [18], and evaluate which co-culture objective (e.g. individual or community growth) best matches experimental data [17], [19].

The idea of using microbial consortia to solve multiple tasks in complex environments has also drawn tremendous attention [20]–[22], and successful examples have illustrated the use of consortia for biotechnological applications [23]–[25]. In addition to these studies, many new tools have been developed to create novel microbial cross-feeding interactions, structured consortia, as well as, quorum-sensing communication [26]–[28]. Creating novel interacting systems allows hypotheses to be tested and reveals ecological principles [29].

Despite these promising findings, the study of microbial consortia has just recently begun and many questions remain. How do species first establish a cooperative community? Does cooperation persist during evolution? When does community or strain fitness increase and what mechanisms drive such improvements? How does the population structure change over time? How do phenotypes of individual strains change during evolution?

To answer these questions, we constructed a synthetic cooperative community of two auxotrophic *E. coli* mutant strains to study how adaptive evolution influences community phenotypes and structure, as well as, individual strain behaviors. In our synthetic community, strain L (which is unable to catalyze an intermediate step in leucine biosynthesis) and strain K (which is unable to catalyze the last step in lysine biosynthesis) can only grow in glucose minimal medium if they exchange leucine (or its precursors) and lysine. The community was adaptively evolved and its growth rate improved by almost three-fold. Monitoring the population dynamics during evolution showed a decrease in the ratio of lysine to leucine auxotrophs over time. Isolates from evolved co-cultures showed improved growth when co-cultured with their un-evolved partner strain compared to the un-evolved K and L co-culture. We additionally used a genome-scale metabolic model of the co-culture to investigate how uptake and release of essential amino acids would influence co-culture growth rate and composition, and hypothesize mechanisms for observed adaptive evolutionary changes. This study provides insights into the evolution of cooperating communities and how microbial phenotypes are altered during adaptive evolution in a co-culture environment. In addition, this study for the first time investigates how individual isolates in the evolved community influence community growth and composition.

## Materials and Methods

### Strains and plasmids

*E. coli* BW25113 and the plasmids pKD46, pCP20, and pKD13 were obtained from *E. coli* genetic stock center. The pKD3 plasmid was provided by Dr. Brian Pfleger (UW, Madison). The *E. coli* knockout strains Δ*leuA::kan*, Δ*lysA::kan*, and Δ*recA::kan* mutants were obtained from the Keio collection (Open Biosystems). An *E. coli* BW25113 Δ*recA::cat* strain was constructed using a PCR-based method [30]. A PCR product was generated that contains the chloramphenicol resistance cassette (*cat*) from pKD3 and has homology to the upstream and downstream sequences of *recA*. The following primers were used in the PCR reaction with pKD3 as a template, 5′- ATGCGACCCTTGTGTATCAAACAAGACGATTAAAAATCTTCGT TAGTTTCGTGTAGGCTGGAGCTGCTTC-3′ and 5′-CAGAACATATTGACTATCCGGTATTACCCGGCATGACAGGA GTAAAAATGCATATGAATATCCTCCTTAG-3′. *E. coli* BW25113 containing pKD46 was transformed with the PCR product using electroporation. Cells were added into 1 mL SOC medium (Fisher Scientific) with addition of 5 mM L-arabinose, incubated at 37°C for 2 hours, and plated on a LB agar plate containing 34 µg/mL chloramphenicol. To generate the double *E. coli* mutants used in the co-cultures, Δ*leuA recA::cat* (referred to as strain L since it is a leucine auxotroph) and Δ*lysA recA::kan* (referred to as strain K since it is a lysine auxotroph), the temperature-sensitive plasmid pCP20 was used to remove the *kan* gene from the BW25113 Δ*leuA::kan* and Δ*lysA::kan* mutants. The Δ*recA::kan* and Δ*recA::cat* mutations were then moved into these two kanamycin sensitive strains by P1 transduction [31] and selected on LB agar plates with kanamycin (50 µg/mL) or chloramphenicol (34 µg/mL).

### Media and culture conditions

Most liquid co-cultures were grown at 37°C in M9 minimal medium (pH 7.0, 100 µM CaCl_2_, 2 mM MgSO_4_, 6.4 g/L Na_2_HPO_4_•7 H_2_O, 1.5 g/L KH_2_PO_4_, 0.25 g/L NaCl, 0.5 g/L NH_4_Cl) supplemented with 2 g/L glucose. For some mono-culture experiments, L-lysine or L-leucine was added into the medium at different concentrations. A concentration of 10 mg/L leucine or lysine was used for the un-evolved strains, since this allowed for significant growth while still ensuring that the amino acid was the limiting nutrient. Higher (16 mg/L) and lower (1.6 mg/L) lysine and leucine concentrations were used to evaluate the evolved isolates in mono-culture, so that growth rates could be measured (for concentrations below 1 mg/L, the change in optical density (OD) during growth was too small to estimate growth rates accurately). For mono-culture experiments, cells were inoculated on LB agar plates with kanamycin (50 µg/mL) or chloramphenicol (34 µg/mL) for 24 hours and resuspended in glucose minimal media. The starting OD600 was 0.01 and 0.05 for un-evolved and evolved strains, respectively. For co-culture experiments, cells from frozen stock were first grown separately in glucose M9 minimal media with 10% (v/v) luria broth (LB) for 24 hours at 37°C, and then pelleted and washed twice using minimal medium without glucose, to remove any residual nutrients from the preculture. Strains were then combined into a co-culture in glucose minimal media.

### Adaptive evolution

Multiple parallel co-cultures of K (lysine auxotroph) and L (leucine auxotroph) strains were each started with a 1∶1 ratio based on OD600 values. Co-cultures were started with an initial OD600 of 0.0065 and were grown in 250 mL flasks containing 100 mL glucose minimal medium. Co-cultures were grown aerobically in a shaking incubator at 37°C. The OD600 of the co-culture was monitored and when it reached ∼0.2 the co-culture was transferred to fresh media (resulting in an OD600 between 0.001 and 0.01) and 3 mL of culture was stored at −80°C. The growth rate of adaptively evolved co-cultures at each passage was approximated using the duration and the change in OD600 value of the passage. The percent of dead cells for the first 5 passages was determined using SYTOX Green nucleic Acid Stain (Molecular Probes, Invitrogen, cat. no. S7020). Frozen co-cultures were later recovered by growing them in 2 mL glucose minimal medium and transferring them into 200 µL of fresh medium (such that the starting OD600 was 0.01) in a 96 well plate and grown at 37°C for 4 days. OD600 values were measured in a Tecan microplate reader and the changes in OD600 values and growth rates for the co-culture were calculated.

### Mono-culture and hybrid co-culture of evolved strains

Evolved isolates from the frozen co-culture samples were obtained by selecting colonies from LB + kanamycin (50 µg/mL) and LB + chloramphenicol (34 µg/mL) agar plates. For mono-culture and hybrid co-culture experiments (consisting of evolved isolates [L^ev^ or K^ev^] and their un-evolved partner strain [K or L]), evolved isolates were grown on LB + kanamycin (50 µg/mL) or LB + chloramphenicol (34 µg/mL) plates and a single colony was used to inoculate cells into glucose M9 minimal medium with (for mono-culture experiments) or without (for hybrid co-culture experiments) leucine or lysine. Mono-cultures and hybrid co-cultures were started with an initial OD600 of 0.05 and 0.01, respectively. Each evolved isolate mono-culture was repeated in triplicate in 384 well plates and grown for 48 hours at 37°C, where OD600 values were measured every 15 minutes. Growth rates were determined by searching for the maximum growth rate in a 3 hour window during exponential growth. A 3 hour window was used because this was less than the exponential growth period for the different cultures and it had enough data points (>10) to get a good estimate for the growth rate. Hybrid co-cultures containing a 1∶1 mixture of evolved isolates and un-evolved K or L strains were carried out in 96 well plates. Hybrid co-cultures were grown in glucose medium at 37°C for 72 hours. A total of four replicates were done, two each on different plates. The OD600 values were monitored every 4 to 6 hours and used to estimate the growth rates.

### Concentration measurements

A bioassay was used to measure the concentration of amino acids. A standard curve for converting a change in OD600 values of strain K to lysine concentrations was generated by growing the K strain (Δ*lysA recA*::*kan*) to stationary phase in glucose minimal medium with various concentrations of lysine for 48 hours. The change in OD600 was proportional to the concentration of lysine, with a proportionality constant of 25.91 mg/L lysine per OD (**Figure S1** in **File S1**). To measure the concentration of lysine in the culture medium, we passed the culture medium through a 0.2 µm nylon membrane to remove cells. The filtrate was then mixed with an equal volume of glucose minimal medium, inoculated with the K mutant and grown at 37°C for 48 hours. The concentration of lysine present in the filtrate was then estimated from the change in OD600 and the proportionality constant.

To estimate the levels of leucine, *Lactobacillus casei* 12A (provided by James L. Steele, UW Madison) was used as a leucine biosensor, since it is incapable of synthesizing leucine. A standard curve was generated using the same method described above, but the growth medium was comprised of equal parts by volume, 2 g/L glucose M9 minimal media with various concentrations of leucine and CDM medium without leucine [32]. The proportionality constant was 20.45 mg/L leucine per OD (**Figure S1** in **File S1**). To quantify the amount of leucine in the culture medium the same procedure described above was used, except *L. casei* was used instead of strain K and the filtrate was mixed with an equal volume of CDM medium without leucine.

The lower limit of detection for leucine and lysine that can be measured accurately using the bioassays was ∼3.5 µM. One limitation of the bioassay is that the filtrate could contain chemicals that inhibit or enhance cell growth causing the bioassay to underestimate or overestimate the amino acid concentrations. To minimize the effects of other chemicals the filtrate was diluted two fold.

Glucose concentrations were measured using a glucose assay from Sigma (GAGO20) after cells were removed using a 0.2 µm nylon filter.

### Estimation of growth and uptake rates

The growth rate and biomass requirements in mono-cultures were estimated from concentration measurements. First, the growth rate (μ) during exponential growth was calculated from the slope of a linear fit between ln(OD) and time (given by lnOD  =  μ⋅t+constant). To estimate the biomass requirements (mmol substrate/gDW) for glucose, lysine, or leucine (*Y_Glc_, Y_Lys_* and *Y_Leu_*), the OD600 values were converted to biomass concentration (g dry weight/L; gDW/L) using a conversion factor of 0.415 gDW/(L⋅OD) [33]. A linear regression between the measured substrate (glucose, leucine or lysine) and biomass concentrations (*X_K_* or *X_L_*) was performed, and the resulting slopes corresponded to the biomass requirements (e.g., [lysine]  =  −*Y_Lys_*⋅*X_K_* + constant). Substrate uptake rates (mmol/gDW/hour) for glucose, lysine and leucine (*U_Glc_, U_Lys_* and *U_Leu_*) in mono-cultures and co-cultures were then estimated by multiplying the measured biomass requirements by the growth rate (**Eq. 1**). Release rates (mmol/gDW/hour) for lysine (*R_Lys_*) and leucine (*R_Leu_*) in co-cultures were estimated by equating the amount of amino acid produced by one strain to the amount consumed by the other strain (**Eq. 2** and **3**).
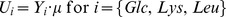
(Eq. 1)

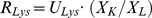
(Eq. 2)

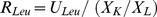
(Eq. 3)


### Quantifying relative populations in the co-culture

Standard plating methods measuring the colony forming units (CFUs) on LB, LB + kanamycin (50 µg/mL) and LB + chloramphenicol (34 µg/mL) agar plates were initially used to quantify the relative abundance of strains K and L in the co-culture (see **Figure S2** in **File S1**). However, the adaptively evolved strains grew poorer on LB plates and the CFUs/(mL⋅OD) decreased. Thus, we decided to use quantitative PCR (qPCR) to determine the relative abundance of the two populations in co-culture based on genomic DNA abundance rather than CFUs. qPCR has been used previously to quantify cellular abundances in co-cultures [26], [34], [35]. While qPCR will quantify both viable and non-viable cells, the results could be affected by differences in chromosome copy numbers. The genomic DNA of 500 µL of the frozen co-cultures was extracted using the Qiagen DNeasy Blood and Tissue Kit (cat. no. 69504). Fragments of the *kan* and *cat* genes (which were located at the same chromosomal position in each strain) were amplified from genomic DNA using qPCR with primers, qkan-L (5′-CTCGTCCTGCAGTTCATTCA-3′), qkan-R (5′-AGACAATCGGCTGCTCTGAT-3′), qcat-L (5′-CGTAATTCCGGATGAGCATT-3′), and qcat-R (5′-TCCGGCCTTTATTCACATTC-3′). Each 20 µL PCR reaction contained 10 µL SsoAdvanced SyBR Green supermix (Bio-Rad), 500 nM forward primers, 500 nM reverse primers and 20 ng genomic DNA. Each assay included triplicates for each co-culture, duplicate no DNA control, and positive controls of 0.1 ng, 1 ng, 10 ng, 100 ng of a 1∶1 mixture containing genomic DNA from the un-evolved K and L strains. The positive controls were used to generate a standard curve. The uncertainty for the estimated DNA concentration using the standard curve was calculated based on the error propagation method [36] (see **File S2** for details).

### Co-culture metabolic model

A dynamic co-culture model was constructed which uses a stoichiometric matrix for each strain (based on the previously published stoichiometric matrix for the iAF1260 model [37]). The fluxes through reactions associated with the deleted genes in the K and L strain were constrained to be zero in the corresponding network. The amount of leucine or lysine consumed by one cell type in the co-culture was constrained to be equal to the amount released by the other cell type. The concentration of glucose, amino acids and biomass were calculated at 0.1 hour intervals using dynamic flux balance analysis (dFBA) [38]. At each time step the dFBA model maximized the combined growth rate of the two strains. The following additional parameters were used in the co-culture model: maximum glucose uptake rate (10 mmol/gDW/hour), lysine and leucine uptake or release rates (varied depending on the condition being simulated), initial concentration of biomass (0.0027 gDW/L), and initial glucose concentration (11 mM). See supplementary methods for additional modeling details in **File S2**.

## Results

To understand how a cooperative community is established and affected by adaptive evolution, we first gathered information about the initial behavior of individual community members in mono-culture and co-culture. In this study, we focused on examining how adaptive evolution affected community growth rates and compositions, and how mono-culture and co-culture phenotypes of individual evolved isolates were affected by adaptive evolution in a co-culture environment. A computational model was then used to evaluate how changes in uptake and secretion rates of cross-fed amino acids would influence community growth rates and composition, and predictions were compared to experimental results.

### Characterization of individual auxotrophs in mono-culture

We first characterized the growth and survival of two auxotrophs in mono-culture to estimate their essential amino acid requirements and to characterize how well they initially survive without their essential amino acids. Lysine (strain K) and leucine (strain L) *E. coli* auxotrophs were used in this work to study microbial interactions in co-culture. To reduce the chance of horizontal gene transfer between the two auxotrophs, we deleted *recA* from Δ*lysA* and Δ*leuA* mutants and replaced it with an antibiotic resistance marker to generate strain K (Δ*lysA recA*::*kan*) and strain L (Δ*leuA recA::cat*). Strain K requires lysine for growth, while strain L requires leucine. The additional deletion of *recA* did not reduce the mutant growth rates compared to the *recA* positive Δ*lysA* and Δ*leuA* mutants (in LB the growth rates were ∼1.32 h^−1^ and ∼1.36 h^−1^ for the *recA* negative and positive strains, respectively).

**Figure 1:**
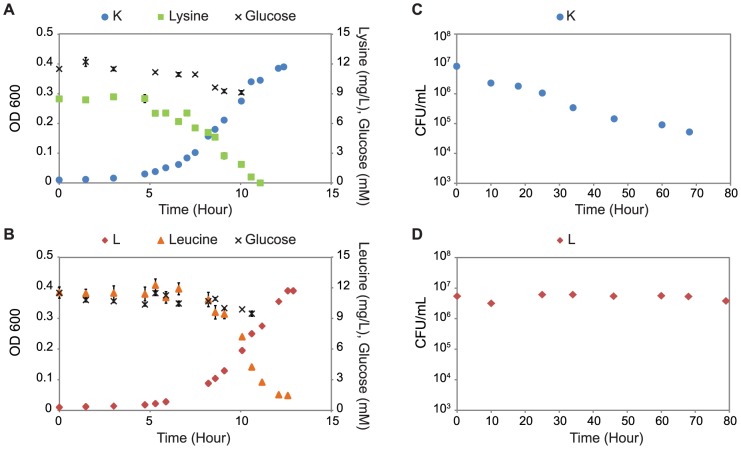
Characterization of mutant growth in mono-culture. (A and B): Un-evolved K and L strains were grown in mono-culture in glucose minimal medium supplemented with 10 mg/L lysine or leucine, respectively. Concentrations of strain K (blue circles), strain L (red diamonds), leucine (orange triangles), lysine (green squares), and glucose (black x) in mono-cultures of strains K and L are shown. (C and D): Survival of strains K (panel C) and L (panel D), in mono-culture in glucose minimal medium without amino acid supplementation. The error bars represent standard deviations across three replicate measurements.

Both the K and L strains were characterized individually in mono-culture during growth in glucose minimal medium when supplemented with lysine and leucine, respectively. When grown in mono-culture where the essential amino acid (lysine or leucine) is limiting, the strains exhibited constant amino acid consumption rates and growth rates (**Figure 1A and 1B**), which were estimated from the concentration data. In mono-culture, strains K and L had similar growth rates; however, strain K had a lower essential amino acid uptake rate than strain L, indicating that *E. coli* needs more leucine than lysine for biomass production. The amino acid requirements were also estimated from the biomass and concentration measurements (**Table 1**, see Methods for details), and they represent the amount of amino acid needed to produce 1 gDW of cells. Specifically, 0.350 mmol of lysine was needed for the formation of 1 gDW of strain K and 0.473 mmol leucine for 1 gDW of strain L. These values are close to the reported biomass composition of *E. coli* B/r, which contains 0.326 mmol lysine and 0.428 mmol leucine per gDW of cells [39]. Accordingly, if these strains have the same growth rate, the leucine uptake rate by L will be higher than the lysine uptake rate by K.

**Table 1:** Mutant phenotypes during growth in mono-culture.

	Δ*lysA* mutant estimated value	Δ*leuA* mutant estimated value
Growth rate (hour^−1^)	0.461	0.465
Amino acid requirement (mmol/gDW)*	0.350	0.473
Amino acid uptake rate (mmol/gDW/hour)‡	0.161	0.220

*The amino acid requirements represent the amount of leucine or lysine required for production of 1 gDW of cells.

‡ The uptake rates are estimated as the product of the growth rate and amino acid requirements.

Since co-cultures of K and L would be carried out without supplementation of leucine and lysine, we also evaluated the survival of strains in mono-culture in glucose minimal medium without addition of amino acids. Cell viability was monitored over time by quantifying the number of colony forming units (CFUs) per mL (**Figure 1C and 1D**) and percent of dead cells using Sytox green nucleic acid stain (**Figure S3** in **File S1**). Interestingly, the two strains showed different resistances to starvation, which has been reported for other amino acid auxotrophs [11], [15]. For the K strain, the number of CFUs/mL decreased within 10 hours, while the L strain did not show a large drop in CFUs/mL over 80 hours.

### Characterization of un-evolved co-cultures

**Figure 2:**
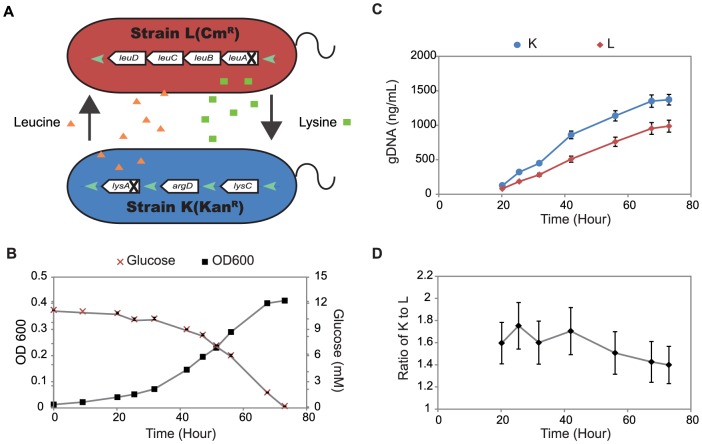
The un-evolved co-culture of strains K and L. (A) Nutrient exchange and dependence in co-culture of two *E. coli* strains L and K. Strain L is incapable of synthesizing leucine, while strain K is unable to synthesize lysine. In the co-culture, if exchange of leucine and lysine occurs then both strains can grow in glucose minimal medium. Panel (B) shows the concentration profiles of glucose (red x) and optical density (black squares) during batch growth of the co-culture. The error bars indicate the standard deviations across replicates. (C) Genomic DNA from the two mutants were extracted from the co-culture at several time points during batch growth of the co-culture and analyzed by qPCR. Blue circles and red diamonds represent the K and L strains, respectively. The error bars were calculated by the error propagation method described in **File S2**. (D) The ratio of K to L was calculated from the qPCR results. The K to L ratio measured using qPCR was 1.59±0.18 for a 1∶1 mixture of un-evolved cells based on OD600. The error bars indicate standard deviations.

We next explored the growth behavior of a co-culture of K and L strains in glucose minimal media (without amino acid supplementation) to see if the K and L strains could exchange essential metabolites. Both mutants were inoculated with the same initial density in glucose minimal medium and growth of the co-culture was monitored over 70 hours. One surprising feature of the co-culture was that there was no lag phase at the beginning of the co-culture, even though the strains were precultured separately. The co-culture had an exponential growth rate equal to 0.056 h^−1^ (**Figure** **2B**), which was around ∼12% of the mono-culture growth rates (**Table 1**). The glucose uptake rate for the co-culture was estimated to be 2.42 mmol/gDW/hour (**Table S1** in **File S1**). We also quantified the relative size of the two mutant populations at different time points of the co-culture by extracting genomic DNA and amplifying the *kan* and *cat* genes using qPCR. In the co-culture, the K and L strains proliferated at very similar rates with L growing slightly slower than K (**Figure 2C**). An equal mixture of K and L (based on OD600 values) corresponds to a K:L ratio (based on genomic DNA levels measured by qPCR) of 1.59±0.18 which is similar to the ratio determined from CFUs (1.54, using data from **Figure 1C and 1D**), and the average K:L ratio determined by genomic DNA levels during exponential growth of the co-culture was 1.6 (**Figure** **2D**). These results indicate that an exchange of leucine (or its precursors) and lysine happened immediately when the two mutants were grown together and was enough to support stable exponential growth.

### Evolution of co-culture

We then adaptively evolved the co-culture for short (∼20 generations) and long (up to ∼167 generations) periods of time to see if we could establish a more cooperative artificial microbial community. The initial co-culture had an exponential growth rate that was 88% lower than the strains grown in mono-culture supplemented with amino acids, so there was significant room for improving growth of the co-culture. We first adaptively evolved three replicate co-cultures for five passages starting with equal amounts (based on OD) of strains K and L in glucose minimal medium. The co-cultures were maintained in prolonged exponential growth by serially transferring cells into fresh medium, and the OD was monitored over the five passages (**Figure S4A** in **File S1**). In all three independent co-cultures, the growth rate was constant over the first two passages (μ∼0.05 h^−1^) and improved by 3-fold during the third passage and then stabilized (μ∼0.14 h^−1^, **Figure S4B** in **File S1**). Interestingly, just like in the initial co-culture the cells did not appear to have any lag phase during the later passages. The average percent of dead cells across the three co-cultures decreased over the first five passages (Spearman Rank Correlation, R^2^ = 1, p = 0.016), ranging between ∼5% and ∼2% at mid-exponential growth (**Figure S5** in **File S1**).

**Figure 3:**
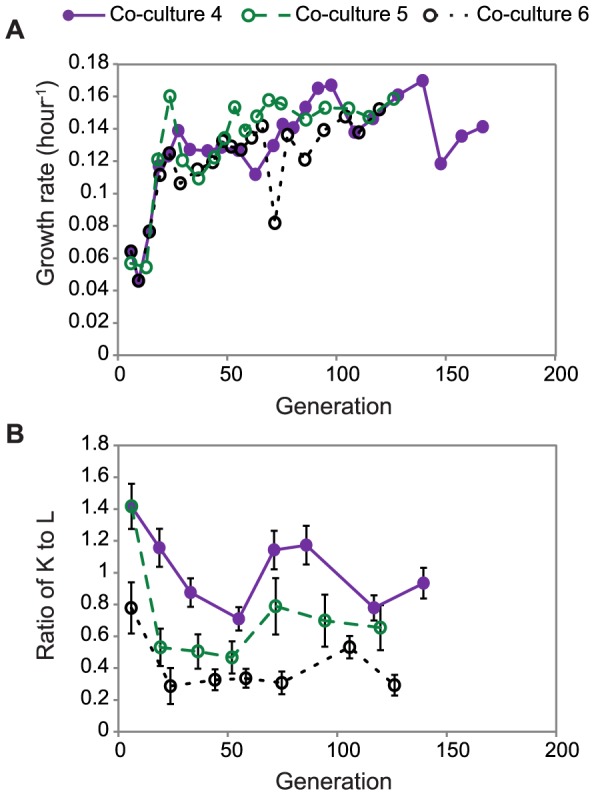
Adaptive evolution of the co-culture. Three parallel co-cultures were performed, represented as a purple solid line (co-culture 4), a green short dashed line (co-culture 5), and a black dotted line (co-culture 6). (A) Growth rates were calculated based on the starting and ending OD values for each passage. (B) Genomic DNA was extracted from frozen samples of the co-culture taken at the end of each passage (OD≈0.2). Relative populations of K and L were estimated using qPCR and used to calculate the ratio of K to L. The error bars represent standard deviations calculated using the method described in **File S2**.

After the short-term evolution experiments, we performed three parallel long-term adaptive evolutionary experiments of the co-culture using the same serial transfer process. The adaptive evolution lasted between 30 and 40 days, and included over 20 passages and over 100 generations. Periodically, a small amount of co-culture was spread on LB agar plates and subsequently transferred to glucose, LB + kanamycin and LB + chloramphenicol agar plates, to check that one strain did not become independent of the other and take over the culture. For these co-cultures, we did not observe any isolates that were able to grow on glucose plates. The growth rate for each passage was estimated from the change in OD values and duration of each passage (**Figure 3A**). Similar to our short-term adaptive evolution results, the growth rate increased around day six in these independent co-culture experiments. After 10 days (5 passages), the growth rates oscillated around the same value. The three parallel co-cultures showed similar endpoint growth rates, which has been observed during evolution of individual strains [40]; however, the evolutionary trajectories of the co-cultures were different, indicated by different growth rates on the same day of evolution.

At the end of each passage, a sample of each co-culture was frozen and stored at −80°C. These frozen co-culture samples were later recovered and further evaluated to study the population dynamics of the co-culture and monitor the evolution of each strain. While the growth rates of the co-culture were higher after evolution, it was unclear if the biomass yields of the evolved co-culture increased in the same fashion since the strains were transferred before reaching stationary phase. When frozen co-cultures were transferred directly into glucose minimal media, the frozen co-cultures tended to grow faster than the fresh co-cultures, which could be due to cell lysis caused by the freeze-thaw process. So we first recovered frozen co-cultures in glucose minimal medium and then passed the exponentially growing culture into fresh medium. Cells were then grown to stationary phase in a microplate reader, allowing growth rates and changes in OD600 values to be quantified (**Figure S6** in **File S1**). In microplates, the change in OD600 of the un-evolved co-culture was 0.26 and all evolved co-cultures showed higher changes in OD600 than the un-evolved co-culture. The growth rate of the un-evolved co-culture was ∼0.05 h^−1^ (similar to the value observed in flask experiments), while the growth rates of evolved co-cultures were 2- to 3-fold faster (**Figure S6** in **File S1**).

In addition to measuring growth phenotypes of the co-culture, we monitored the relative abundance of the two strains over adaptive evolution. To estimate the relative abundance of the two strains at the end of each passage, genomic DNA from the frozen co-culture was extracted and qPCR was used to estimate the cell ratios (**Figure 3B**). The ratio of K to L decreased in all three evolved co-cultures and the final K:L ratios varied across the different parallel co-cultures between 0.93 and 0.29. The lower K:L ratio indicates that a smaller population of K cells can maintain a larger population of L strains. This could be due to a higher release rate of leucine (or its precursors) via secretion or cell lysis compared to lysine or a higher uptake rate of leucine compared to lysine. Since we did not detect any leucine or lysine in the co-culture medium, we cannot exclude either possibility.

### Characterization of evolved strains in mono-culture

During adaptive evolution, co-cultures of K and L strains achieved higher growth rates and biomass yields. However, these experiments were done with a heterogeneous population and not using individual isolates. To further investigate how adaptive evolution affected individual strain behaviors we isolated strains from different passages of the co-culture and evaluated their growth in mono-culture and then in co-cultures (described in next section). We randomly selected colonies of evolved K (or L) strains from different passages in co-culture 4 and 6 (since these co-cultures were the most different), and grew individual evolved isolates (K^ev^ and L^ev^) in mono-culture in glucose minimal medium with different concentrations of lysine or leucine.

**Figure 4:**
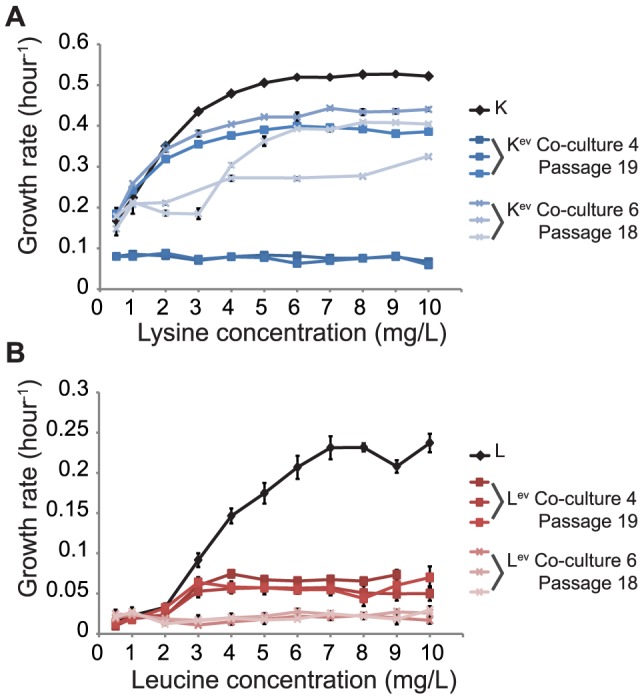
Mono-culture of K^ev^ and L^ev^. Three randomly selected colonies of K^ev^ (or L^ev^) from passage 19 of co-culture 4 and passage 18 of co-culture 6 were inoculated in glucose minimal medium with various amounts of lysine (for K^ev^ strains, panel A) or leucine (for L^ev^ strains, panel B). Each colony was tested in three replicate mono-cultures. The growth rates were calculated for the evolved and un-evolved parental strains (control). The error bars represent the standard deviations across biological replicates.

We first selected 3 colonies of K^ev^ (or L^ev^) from late passages of co-cultures 4 and 6 and inoculated them in medium with increasing amounts of lysine or leucine. Surprisingly, the K^ev^ and L^ev^ strains had lower growth rates and changes in OD600 values compared to the un-evolved K and L strains, except for some K^ev^ isolates in co-culture 6 which had higher changes in OD600 values (**Figure 4** and **Figure S7** in **File S1**). We subsequently evaluated 10 isolates from different passages of co-cultures 4 and 6 for growth in mono-culture in the presence of high (16 mg/L) and low (1.6 mg/L) concentrations of lysine or leucine. In general, we found that some isolates from earlier passages did show improved growth phenotypes in high and low concentrations of amino acids, but that most isolates from later passages had decreased growth rates and changes in OD600 values than the un-evolved K and L strains (**Figure S8** in **File S1**).

In addition to studying individual isolates in mono-culture with exogenous amino acid supplementation, we also evaluated the survival of isolates in mono-culture without exogenous lysine and leucine (where they are unable to grow) by measuring the percent of dead cells after 24 hours in glucose minimal medium. Compared to the un-evolved K strain, the evolved K^ev^ isolates from co-culture 4 and 6 had a lower percentage of dead cells (**Figure S9** in **File S1**). On the other hand, the evolved L^ev^ isolates from both co-cultures had a higher percentage of dead cells compared to the un-evolved L strain. These data indicate that possible mechanisms for improving growth of the co-culture could be due to a decreased viability of the L strain and/or increased viability of strain K.

### Properties of evolved isolates in hybrid co-culture

Since the strains were evolved in co-culture and not mono-culture we also sought to evaluate changes in growth phenotypes of individual isolates when grown in co-culture with their un-evolved partner strains (referred to here as a hybrid co-culture). This was done so that we can identify which evolved subpopulations of K and/or L strains are responsible for improving growth of the evolved communities. To find out how evolved isolates derived from each strain affect growth of the co-culture, we evaluated hybrid co-cultures containing evolved isolates (K^ev^ or L^ev^) with their un-evolved partner strains (L or K) in glucose minimal media. The growth rates and biomass yields of L^ev^ + K (or L + K^ev^) hybrid co-cultures were then compared to those of the initial un-evolved co-culture (L+K).

**Figure 5:**
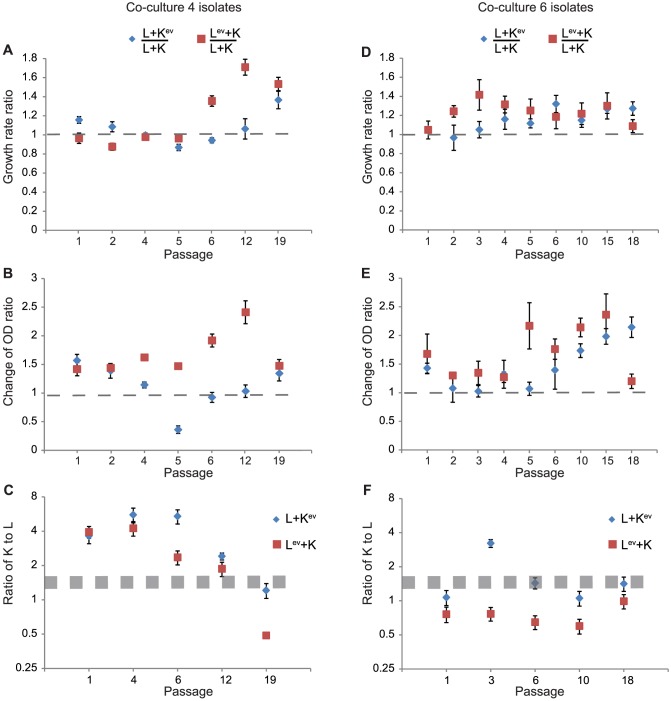
Comparisons between un-evolved co-cultures L+K and hybrid co-cultures containing L+K^ev^ or L^ev^+K. Cells from 10 colonies of K^ev^ (or L^ev^) at each selected passage were grown individually in co-culture with the un-evolved partner strain (L or K). The growth rate and change in OD600 for each hybrid co-culture was normalized to the growth rate and change in OD600 of the un-evolved co-culture grown on the same microplate. The resulting growth rate ratios and change in OD600 ratios are shown as blue diamonds (L+K^ev^) and red squares (L^ev^+K), respectively, in panels A and B (isolates from co-culture 4) and panels D and E (isolates from co-culture 6). The error bars indicate the standard deviations based on 10 separate hybrid co-cultures each with four replicates (n = 40). The dashed lines indicate the behavior of the un-evolved co-culture (L+K). Panels C and F shows the K:L ratio in L+K^ev^ and L^ev^+K in hybrid co-cultures and the un-evolved co-culture. The hybrid co-cultures contained evolved isolates from co-culture 4 (panel C) or co-culture 6 (panel F). The error bars indicate the standard deviations based on hybrid co-cultures using three different isolates and three measurements for each passage (n = 9), see **File S2** for details. The shaded bands in C and F show the mean ± the standard deviation for the K:L ratio in the un-evolved co-culture at an OD600 of 0.2 when grown in 96 well plates (1.62±0.14).

In co-culture 4, the growth rates of L+K^ev^ and L^ev^+K hybrid co-cultures containing isolates from the first five passages were similar to the initial co-culture (K+L) (**Figure** **5A**), while increased growth rates were observed in hybrid L+K^ev^ and L^ev^+K co-cultures containing isolates from later passages. Growth rate improvements in the hybrid co-cultures were slightly delayed compared to our earlier analysis of the evolved co-culture (**Figure** **S4B** in **File S1**), where the biggest growth rate improvements happened after three passages. This delayed improvement in growth rate could be due to the fact that only single evolved isolates were evaluated (rather than a mixed population) and that evolved isolates were tested in combination with un-evolved partner strains (rather than evolved partner strains). Compared to co-culture 4, isolates from co-culture 6 (**Figure 5D**) had larger variations across isolates from the same passage and earlier increases in growth rates. Interestingly, none of the evolved isolates co-cultured with their un-evolved partner strains led to a three-fold improvement in growth rate as observed in the evolved co-culture, indicating that synergistic effects between evolved isolates may exist in the co-culture. In both co-culture 4 and 6, the growth rate of L^ev^+K hybrid co-cultures increased faster than the corresponding L+K^ev^ co-cultures, indicating that the L strains adapt more quickly to enhance co-culture growth. Hybrid co-cultures containing evolved isolates from co-culture 4 and 6 also exhibited higher biomass yields (measured by changes in OD600, **Figure 5B and 5E**).

Since each hybrid co-culture contains at least one of the un-evolved parental strains, if the L+K^ev^ (or L^ev^+K) co-culture grows better than L+K co-culture, then the evolved isolates likely have increased uptake and/or release of leucine (or its precursors) or lysine. An improved uptake rate would increase the abundance of the evolved strain in the co-culture while a higher release rate would benefit its partner strain. The ratio of K:L in the hybrid co-cultures during exponential growth was also measured using qPCR of genomic DNA, and compared to the K:L ratio in the un-evolved co-culture. These ratio measurements allowed us to find out which strain if any dominated the hybrid co-culture. Three hybrid co-cultures at five different passages were selected for this analysis. They represent the slowest, medium and fastest growing hybrid co-cultures within a given passage. For comparison, the co-culture of un-evolved strains (L+K) was measured and had a K:L ratio 1.62±0.14. With isolates from co-culture 6, the K:L ratio of L+K^ev^ hybrid co-cultures at mid-exponential growth were all less than 1.6 (except for passage 3) indicating that the K^ev^ strains improved growth of the L strain more than the original K strain (**Figure 5F**). In addition, the K:L ratios in L^ev^+K hybrid co-cultures also showed a decreasing trend, implying that the L^ev^ strains became dominant in the hybrid co-cultures. These results suggest that the K^ev^ strains may increase release of leucine (or its precursors) and/or the L^ev^ strains increase uptake rates of leucine. The hybrid co-culture with isolates from co-culture 4 showed a very different pattern. The K:L ratio initially increased for both of L+K^ev^ and L^ev^+K hybrid co-cultures compared to the L+K un-evolved co-culture, suggesting possible better exchange of lysine, while the K:L ratio decreased at later passages, suggesting a better exchange of leucine (or its precursors) (**Figure 5C**).

### Model predictions of batch co-cultures

A number of possible mechanisms associated with amino acid exchange could explain the improvements in growth of the co-culture over adaptive evolution. These include increased uptake or release rates of leucine (or its precursors) or lysine, or combinations of these. Direct measurements of cross-feeding rates could not be made, so metabolic modeling was used to gain additional insights. We developed a computational model of the co-culture using a genome-scale metabolic model of *E. coli* [37]. This model was used to evaluate how uptake/secretion rates of essential amino acids would affect co-culture growth rates and community compositions, and to see if the model could predict co-culture behaviors. Dynamic flux balance analysis (dFBA) simulations were performed where the uptake/release of leucine and lysine were varied and the growth rates and K:L ratios were predicted at an OD600 of 0.2. At each time step in dFBA, metabolism was assumed to be at a steady-state and a flux distribution maximizing the combined growth rate was found. Since we did not detect any leucine or lysine in the co-culture media, we additionally constrained the dFBA model to ensure that there was no net accumulation of leucine or lysine in the media.

**Figure 6:**
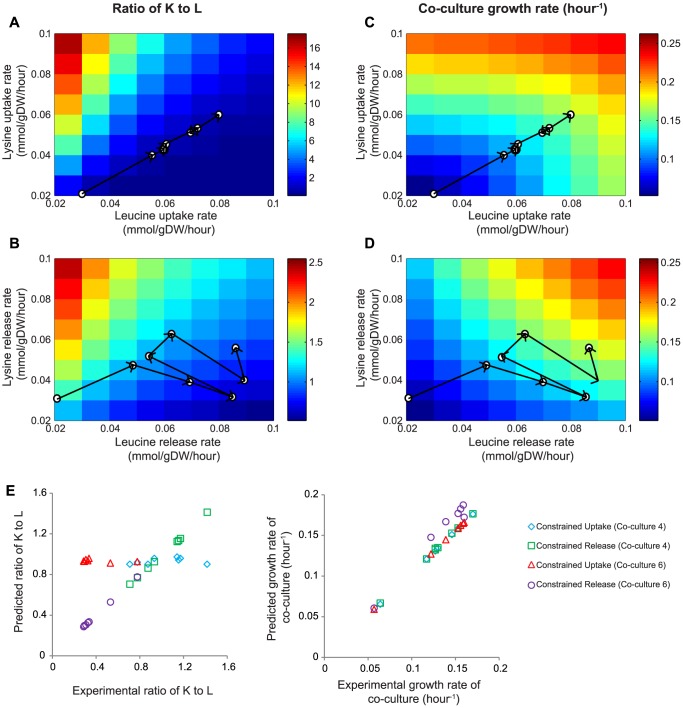
Computational model predictions of co-culture composition and growth rates. The model was constrained using either amino acid uptake (panels A and C) or release rates (panels B and D). Panels A and B display the predicted K:L ratio at a co-culture OD≈0.2. The color map indicates the value of K:L ratio. Panels C and D show the predicted average growth rate of co-culture, indicated by the color map. The evolutionary trajectory of co-culture 4 is shown on panels A through D, where the open circles indicate passages 1,4,7,10,12,15,19 and 21. The estimated uptake or release rates for evolved K^ev^ and L^ev^ strains in each passage were then used to constrain the model. Panel E compares the model predicted K:L ratio and average growth rate of the co-culture near OD600≈0.2 to the estimated experimental values. Blue diamonds and red triangles denote the predictions when the model was constrained by the estimated uptake rates for co-culture 4 and 6, respectively. Green squares and purple circles denote the predictions when the model was constrained by the estimated release rates for the two co-cultures for co-culture 4 and 6, respectively.

As expected, changing the uptake and release rates of the essential amino acids affected the community composition and the average growth rate (**Figure 6**). The model predicted that higher uptake or release rates of lysine will result in a larger K:L ratio, while larger rates of leucine uptake or release will decrease the ratio (**Figure 6A and 6B**). What we did not anticipate is that the strain ratio was predicted to be more sensitive to the uptake rates than release rates. The strain ratio ranged between 0.04 and 17.54 when consumption rates were constrained, compared to 0.26 and 2.5 when release rates were constrained to the same range of values. The growth rate of the co-culture was predicted to improve by increasing uptake and/or release of leucine or lysine (**Figure 6C and 6D**).

A major obstacle in studying the co-culture is an inability to directly measure the real-time uptake and release rates of the exchanged amino acids. We estimated the uptake and release rates of leucine (or its precursors) and lysine for the evolved K (or L) strains using the measured growth rates, biomass requirements and K:L ratios (assuming amino acid requirements did not change, see Methods for details). These estimated uptake and release rates (**Table S2** in **File S1**) were used to project the evolutionary trajectories for co-cultures 4 (**Figure 6**) and 6 (**Figure S10** in **File S1**). The estimated uptake and release rates of leucine and lysine both increased in co-culture 4, while only leucine exchange increased dramatically in co-culture 6. Using the estimated uptake or release rates as inputs, the model was then used to predict the K:L ratio and average co-culture growth rate. The experimentally measured K:L ratios and growth rates in the evolved co-culture were highly correlated to model predictions when release rates were constrained, but not uptake rates (**Figure 6E**). Since the uptake rate was estimated by multiplying the growth rate with the lysine (or leucine) requirement per gDW cells (**Table 1**), constraining the uptake rates effectively constrains the model growth rates to be close to the measured values, resulting in a K:L ratio always close to 1.

## Discussion

We built a synthetic cooperating system with two *E. coli* auxotrophs (Δ*lysA recA::kan* and Δ*leuA recA::cat*) and adaptively evolved the co-culture. In their initial encounter, both strains grew, implying they exchanged leucine (or its precursors) and lysine. Replicate co-cultures all maintained persistent cooperation during adaptive evolution and achieved similar growth rates but resulted in different population compositions and evolutionary trajectories. The experimental data and computational model predictions suggested that one evolved co-culture benefited from a better exchange of leucine (or its precursors), while another evolved co-culture experienced better exchange of both amino acids. Interesting, the timing needed to improve co-culture growth rates (∼10 days) was similar to a previous study on evolving individual strains [41]. Our results showed immediate cooperation between strains in co-cultures, adaptive evolution improved strains' growth in co-cultures and evolved strains had decreased growth in mono-culture and altered ability to survive starvation.

### Cooperation between strains

*E. coli* does not normally secrete amino acids, and amino acid synthesis is well controlled by regulatory mechanisms so that the cellular inputs are best used for growth. In previous studies of auxotrophs, starvation led to cell death and release of some metabolites (amino acids and nucleic acids) [11], [15]. In lysine-limiting media, a Δ*lysA E. coli* mutant (lacking diaminopimelate decarboxylase) has been shown to secrete various metabolites, including diaminopimelate (DAP), an important cell wall constituent [42], [43]. In our study, we observed that in mono-culture without amino acid supplementation, the L and K strains showed different death rates. Given the different death rates of the two strains we expected to see an initial one-way cross-feeding from K to L (not cooperative) and a lag phase prior to exponential growth in co-culture. However, we found reproducible growth of both strains in co-culture and an absence of a lag phase in replicate co-cultures, indicating consistent two-way cross-feeding of leucine (or its precursors) and/or lysine.

We used a genome-scale metabolic model to get a better understanding of how metabolite uptake/release rates affect growth rates of the co-culture (**Figure 6** and **Figure S10** in **File S1**). In general, increases in both release and uptake rates will enhance proliferation of strains and alter community composition. A prior study by Wintermute and Silver developed models to evaluate invested benefits and cooperation levels in *E. coli* co-cultures [10], [44]. They found that when one strain overshares (i.e., is highly cooperative), the other strain becomes dominant in the co-culture. The oversharing strain can only improve its growth if its partner cooperates. Our computational results (**Figure 6B and 6D**) are consistent with these findings. When leucine (or lysine) release is higher in strain K (or L), its corresponding partner strain L (or K) dominates. When its partner strain produces more lysine (or leucine), K (or L) will begin to increase its relative population in the community.

### Altered viability during starvation

Un-evolved K and L strains exhibited different survival rates during lysine and leucine starvation (**Figure 1**). We observed that the un-evolved K strain (Δ*lysA recA::kan*) quickly underwent cell death in the absence of lysine. Cell death and lysis were also observed in a yeast *lysA* mutant [15]. In a previous study of *E. coli* co-cultures, a Δ*lysA* mutant behaved as a universal cooperator, supporting growth of a variety of other auxotrophs in co-culture, while other strains (including Δ*leuB*, Δ*leuC*, and Δ*leuD* mutants) grew with a smaller number of partner strains [10]. Based on our results, cell death could explain how universal cooperators enable co-culture growth through the release of many different metabolites by cell lysis. Another previous study suggests that evolution of cooperative cross-feeding requires an initial one-way cross-feeding by one species [16]. The stability of our K and L cooperative system could be due to strain K's ability to cross-feed metabolites due to cell death.

We additionally observed that the evolved K^ev^ and L^ev^ isolates displayed altered survival during amino acid starvation. The K^ev^ strains adapted to reduce their death rates during lysine starvation, while evolved L^ev^ strains died more quickly during leucine starvation. Increased cell death by L^ev^ strains and decreased cell death by K^ev^ strains could contribute to better metabolite exchange and improvement of the co-culture.

### Reduced growth in mono-culture

In single species adaptive evolution experiments, evolved strains can exhibit improved growth in one environment and reduced growth in another environment. *E. coli* strains evolved in glucose media can lose their ability to utilize other carbon sources [45]. Other strains adapted to low temperatures may have reduced fitness at higher temperatures [46]. The environment in the co-culture is complex, and strains adapted to the co-culture might not grow as well in mono-culture. Our experiments demonstrated that evolved L^ev^ and K^ev^ isolates were able to improve growth of co-cultures (**Figure 5**), but had reduced growth in mono-culture when supplemented with their essential amino acids (**Figure 4** and **Figures S7** and **S8** in **File S1**). Assimilation of amino acids is important for improving co-culture growth and the reduced growth in mono-culture was unexpected. It may imply that strains in evolved co-cultures become dependent on other strains and/or that additional metabolites are being exchanged. Growth in mono-culture could decrease due to a downregulation or loss of essential genes, whose biological roles are fulfilled by the other strain in co-culture. This has been recently referred to as the black queen hypothesis [3]. Further investigation of these evolved strains using gene expression analysis and genomic sequencing could potentially identify genetic reasons for the observed changes in co-culture and mono-culture phenotypes.

In this study, we performed a series of experiments to investigate the behaviors of un-evolved and evolved co-cultures and how individual evolved isolates contribute towards improving co-culture growth. Metabolite (lysine and leucine or its precursors) cross-feeding is essential for co-culture growth but unfortunately could not be quantified directly. Estimated uptake and release rates of essential metabolites increased over adaptive evolution, except for lysine release rates in co-culture 6. In addition to genome and mRNA sequencing, future experimental approaches enabling the direct measurement of nutrient exchange rates in co-cultures would aide in pinpointing the mechanism(s) for the observed growth rate improvements. While this study and others [9]–[13], [15] have focused on cooperative interactions, the adaptive evolution of communities with other types of interactions (e.g., commensalism, amensalism or parasitism) would be of interest as well [24].

## Supporting Information

File S1 Figures S1–S10 and Table S1.
(PDF)

File S2 Supplementary methods with additional details on the metabolic co-culture model and methods for uncertainty propagation using standard curves for quantitative PCR.
(PDF)
