# Supplementary material for: Adaptive Evolution of Synthetic Cooperating Communities Improves Growth Performance
Source: PLoS One. 2014 Oct 9;9(10):e108297. doi: 10.1371/journal.pone.0108297 (PMC4191979; doi:10.1371/journal.pone.0108297)
Supplement: File S1 — Figures S1–S10 and Table S1. (PDF) [file pone.0108297.s001.pdf]

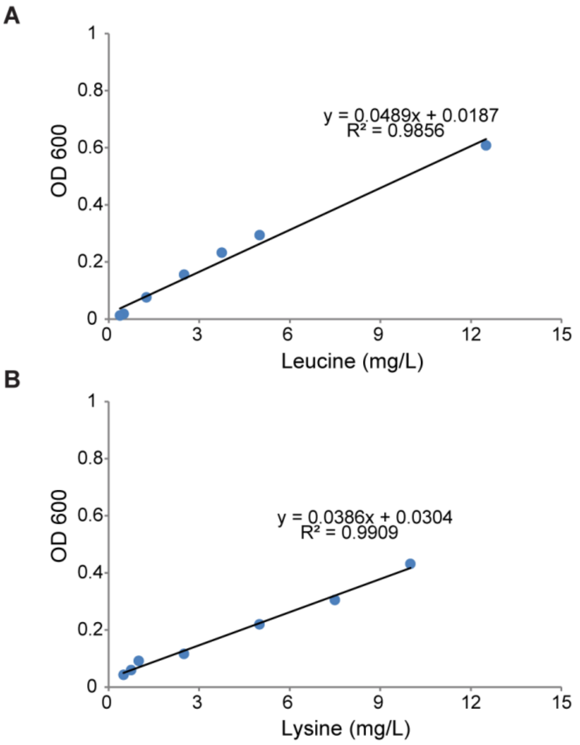

Figure S1. Standard curves for amino acid bioassays. (A) The standard curve for leucine concentration versus OD600 values for *L. casei*. (B) The standard curve for lysine concentrations versus OD600 values for strain K. Tests were performed in 96 well plates and OD600 values were monitored every 15 minutes in microplate reader. The OD values were then converted to OD600 values in a standard spectrophotometer with 1cm pathlength using a standard curve (not shown). Each dot shows the amino acid concentration versus the converted OD600 value minus the OD600 value when the strains were grown in medium without amino acids.

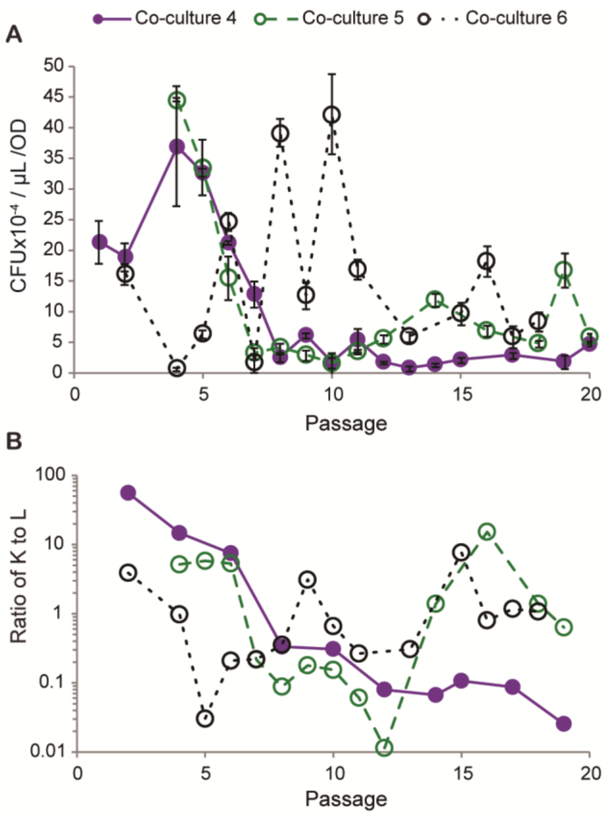

Figure S2. The number of viable cells in co-cultures was quantified by measuring the colony forming units (CFUs) on LB agar plates (A). Error bars indicate standard deviations. The relative abundance of strain K and L in co-cultures were quantified by measuring CFUs on LB+kanamycin and LB+chloramphenicol agar plates and the ratio of K to L was then calculated (B).

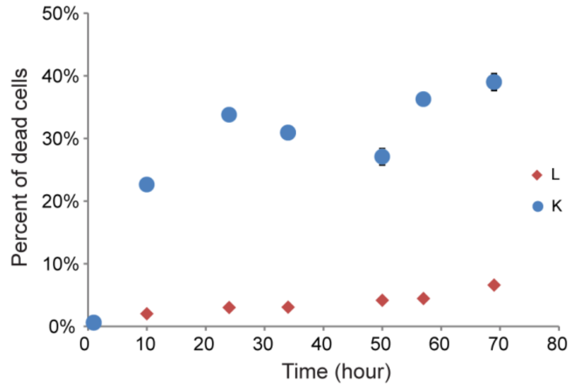

Figure S3. The percent of dead cells in the mono-culture in minimal glucose medium without supply of amino acids. They were measured using Sytox green nucleic acid stain. The error bars indicate standard deviations.

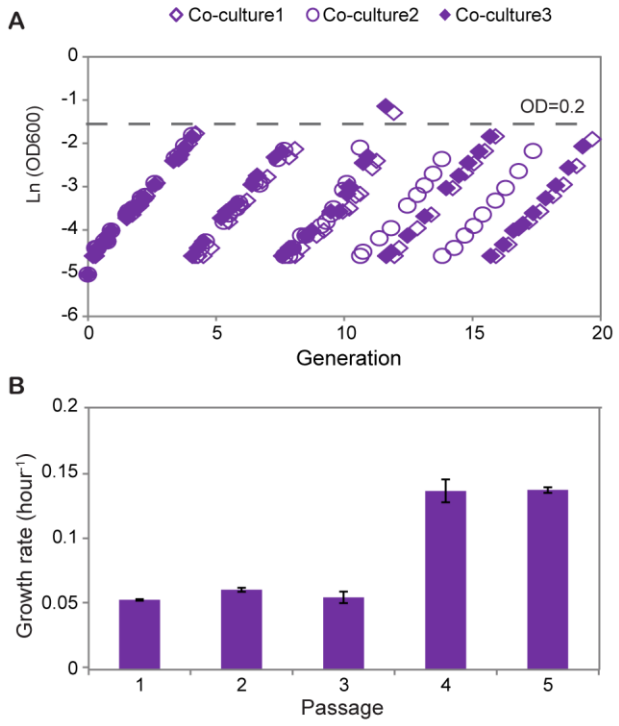

Figure S4. Changes in growth behavior over short adaptive evolutionary time frames. (A) Natural log of the OD over time for three parallel co-cultures (co-cultures 1, 2, and 3) for five passages. Cultures were passed to fresh medium at OD600 ≈ 0.2 (marked as dashed line). The open diamond, open circle and solid diamond denote co-culture1, 2, and 3, respectively. (B) Average growth rates of each passage across the three independently evolved co-cultures. The error bars represent the standard deviations of the growth rate over the three co-cultures.

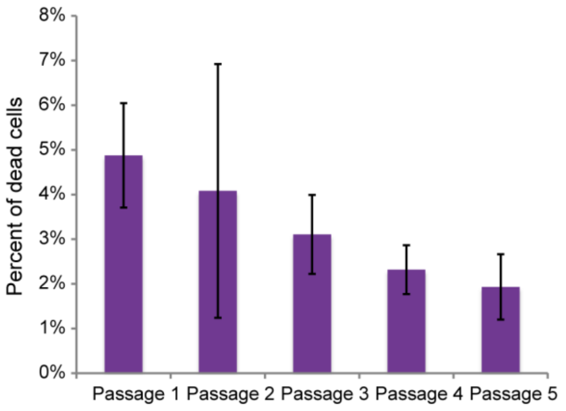

Figure S5. Percent of dead cells in co-cultures. Percent of dead cells in five different passages from co-cultures 1, 2, 3 were measured when OD≈0.2. Each column represents the average percent of dead cells from the three co-cultures. The error bars indicate standard deviations.

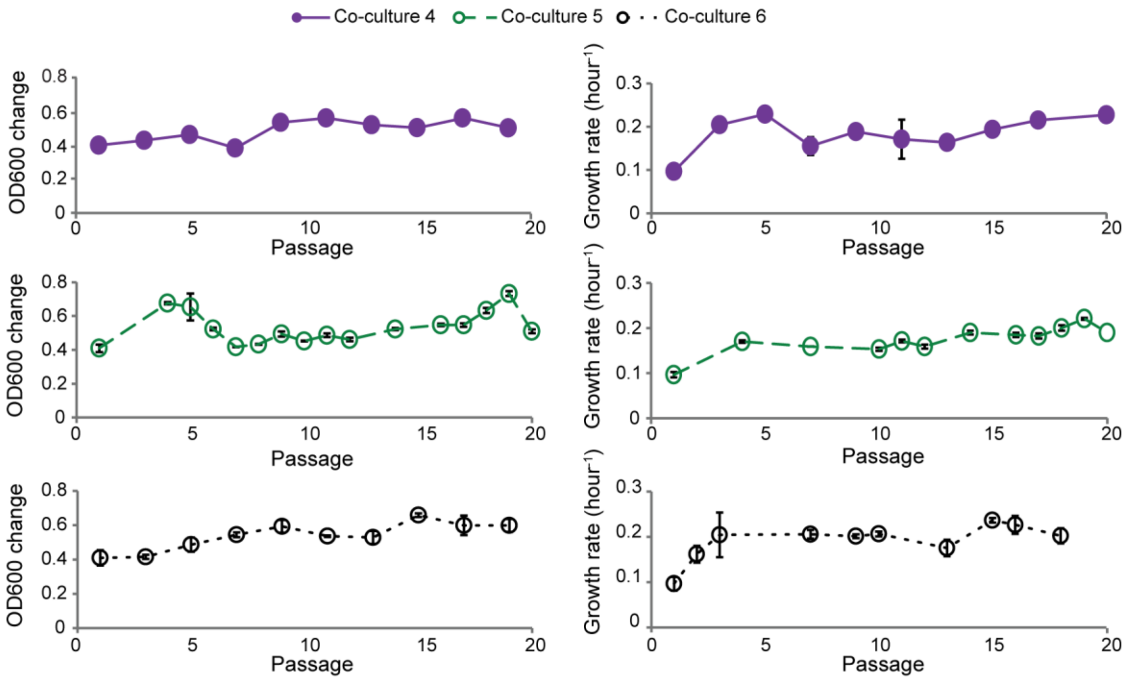

Figure S6. The change in OD600 and growth rates of evolved co-cultures 4, 5 and 6. Frozen co-cultures were recovered and then grown in glucose minimal medium to stationary phase in 96 well plates. The change in OD600 shown in the figure was maximum OD600 value minus the initial value. The unevolved co-culture had a growth rate ~0.05 ($hour^{-1}$) and OD600 change ~ 0.26 in 96 well plates. Co-cultures 4, 5 and 6 are denoted by solid line, dashed line and dotted lines, respectively. The error bars indicate standard deviations.

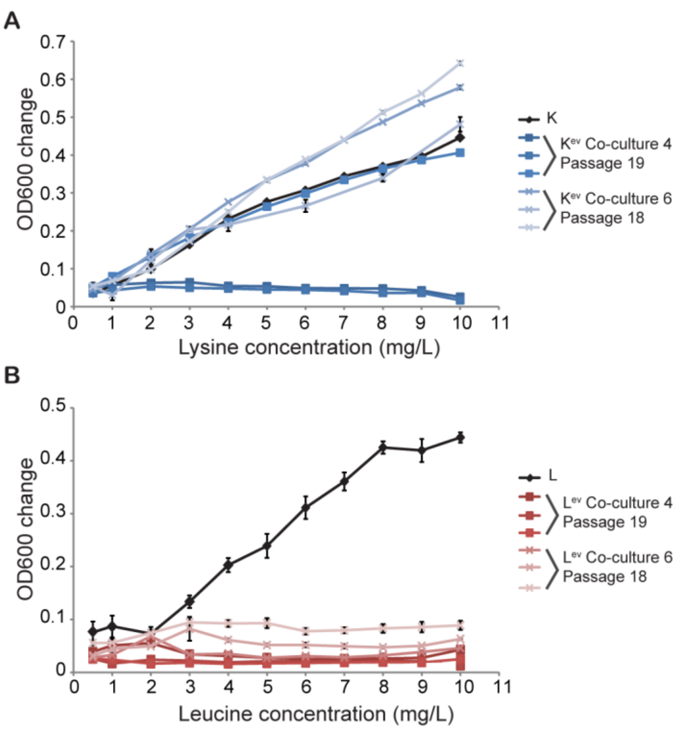

Figure S7. The change in OD600 of evolved isolates (K^{ev} or L^{ev}) or un-evolved strains (K and L) in glucose minimal medium with different concentrations of lysine (panel A) or leucine (panel B). Evolved strains were isolated from passage 19 of co-culture 4 and passage 18 of co-culture 6. All strains were grown in mono-cultures with three biological replicates. The error bars indicate standard deviations.

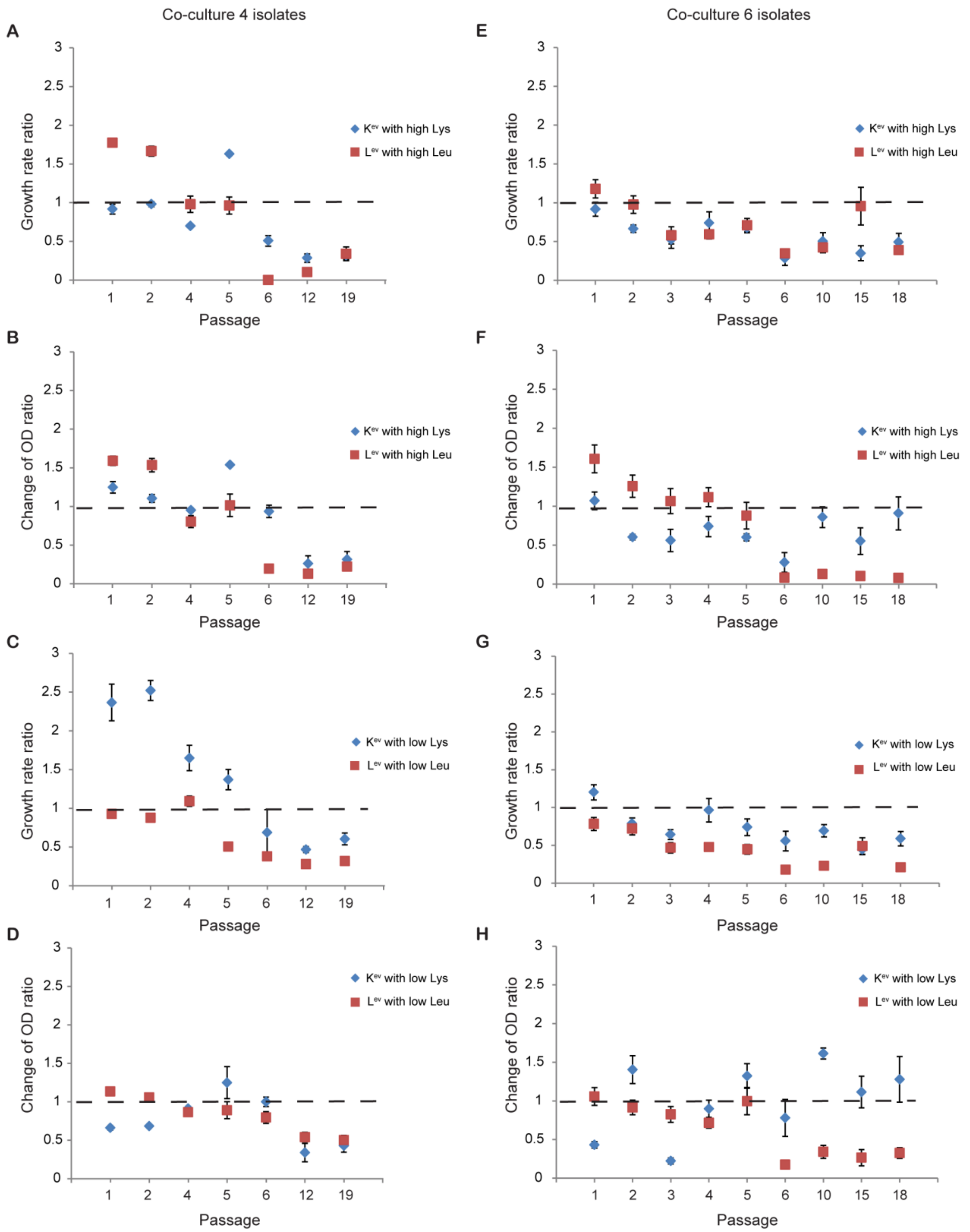

Figure S8. Growth phenotypes of K^{ev} and L^{ev} isolates grown in mono-culture with high and low levels of amino acids. Ten randomly selected K^{ev} (or L^{ev}) isolates from different passages were inoculated in glucose minimal medium supplemented with Lys (or Leu) at high (16 mg/L, panels A, B, E and F) and low (1.6g/L, panels C,D, G and H) concentrations. Each isolate was tested in three replicate mono-cultures in 384 well plates. The growth rates and changes in OD600

were calculated for evolved isolates and normalized to the values for their respective un-evolved parental strains (K or L). The mean of the growth rate ratios and change in OD600 ratios are shown as blue diamond (K^{ev}/K) and red square (L^{ev}/L), respectively, in panels A-D (Co-culture 4) and E- H (Co-culture 6). The error bars indicate standard deviations.

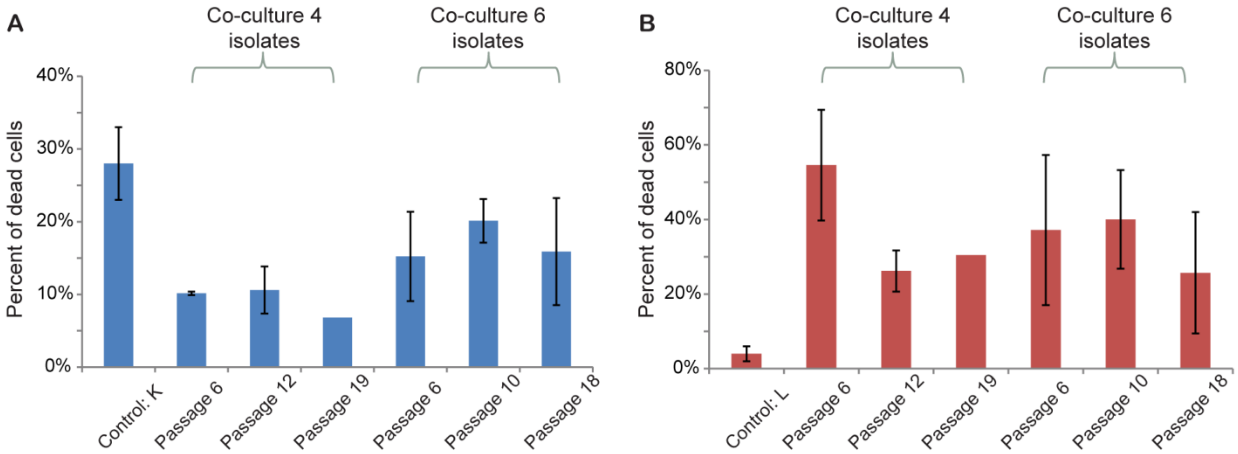

Figure S9. The percent of dead cells in mono-culture. Three evolved K^{ev} (panel A) or L^{ev} (panel B) isolates from different passages were grown in mono-culture in glucose minimal medium. The number of dead cells was measured using Sytox green nucleic acid stain at 24 hour. The control is the percent of dead cells in mono-culture containing un-evolved K or L strains. The bar indicates the standard deviation across replicates.

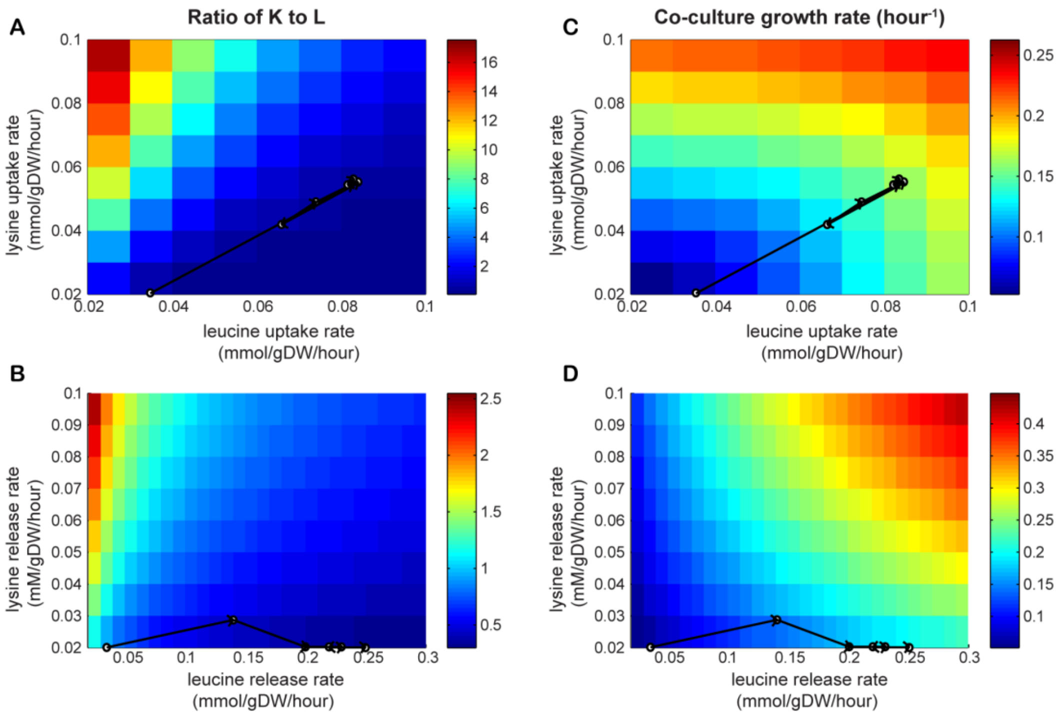

Figure S10. Computational model predictions of co-culture composition and growth rates. The model was constrained using either amino acid uptake (panels A and C) or release rates (panels B and D). Panels A and B display the predicted K:L ratio at a co-culture OD≈0.2. The color map indicates the predicted K:L ratio. Panels C and D show the predicted average growth rate of co-culture, indicated by the color map. The evolutionary trajectory of co-culture 6 is shown on panels A through D, where the open circles indicate passages 1,4,7,10,13, 16 and 18.

Table S1. Glucose uptake rates of evolved and un-evolved co-cultures

	Unevolved Co-culture	Passage 12 of Co-culture 4	Passage 15 of Co-culture 6
Growth rate ($hour^{-1}$)	0.056	0.155	0.161
Glucose requirement (mmol/gDW/hour)	43.288	37.356	37.752
Glucose uptake rate (mmol/gDW/hour)	2.424	5.805	6.059

Growth rates and glucose requirements were calculated from the biomass and glucose concentrations and used to calculate the glucose uptake rate.

Table S2. Estimated uptake and release rates of lysine and leucine during adaptive evolution.

Passage	Lysine uptake rate (mmol/gDW/hour)	Leucine uptake rate (mmol/gDW/hour)	Lysine release rate (mmol/gDW/hour)	Leucine release rate (mmol/gDW/hour)
**Un-evolved Co-culture**				
Un-evolved	0.020	0.027	0.020	0.027
**Co-culture 4**				
1	0.022	0.030	0.032	0.021
4	0.041	0.055	0.047	0.048
7	0.044	0.060	0.039	0.069
10	0.044	0.060	0.032	0.085
12	0.046	0.061	0.052	0.054
15	0.054	0.072	0.063	0.062
19	0.051	0.069	0.040	0.089
21	0.060	0.080	0.056	0.086
**Co-culture 6**				
1	0.020	0.027	0.016	0.035
4	0.056	0.076	0.030	0.142
7	0.043	0.058	0.013	0.197
10	0.049	0.066	0.014	0.229
13	0.055	0.074	0.018	0.226
16	0.054	0.072	0.018	0.215
18	0.056	0.075	0.017	0.245

The uptake and release rates of lysine and leucine were estimated using **equations 1-3** in methods section.
