# Supplementary material for: Adaptive Evolution of Synthetic Cooperating Communities Improves Growth Performance
Source: PLoS One. 2014 Oct 9;9(10):e108297. doi: 10.1371/journal.pone.0108297 (PMC4191979; doi:10.1371/journal.pone.0108297)
Supplement: File S2 — Supplementary methods with additional details on the metabolic co-culture model and methods for uncertainty propagation using standard curves for quantitative PCR. (PDF) [file pone.0108297.s002.pdf]

SUPPLEMENTARY METHODS

Dynamic metabolic model of co-culture

At each time step in a dynamic flux balance model (dFBA) the metabolic model(s) are assumed to be at steady state and flux balance analysis (FBA) is used to predict the fluxes in that time step. Here, a FBA problem was formulated and solved to find the flux distributions in strain K ($\boldsymbol{v}^{K}$), strain L ($\boldsymbol{v}^{L}$), and media concentration rate of change ($\boldsymbol{v}^{Media}$). The units for the fluxes ($\boldsymbol{v}^{K}$ and $\boldsymbol{v}^{L}$) are mmol/gDW/h (except for the biomass flux which is 1/h) and the units for the concentration rate changes ($\boldsymbol{v}^{Media}$) are mmol/L/h. The FBA problem is shown below in Equations 1-9.

max $\boldsymbol{c}^{K} \cdot \boldsymbol{v}^{K} + \boldsymbol{c}^{L} \cdot \boldsymbol{v}^{L}$ Eq 1

such that

$$\boldsymbol{S} \cdot \boldsymbol{v}^{K} = 0 \quad \text{Eq 2}$$

$$\boldsymbol{S} \cdot \boldsymbol{v}^{L} = 0 \quad \text{Eq 3}$$

$$\boldsymbol{LB}^{K} \leq \boldsymbol{v}^{K} \leq \boldsymbol{UB}^{K} \quad \text{Eq 4}$$

$$\boldsymbol{LB}^{L} \leq \boldsymbol{v}^{L} \leq \boldsymbol{UB}^{L} \quad \text{Eq 5}$$

$$v_{i}^{Media} = -\sum_{j} S_{i,j} \cdot \left(v_{j}^{K} \cdot x_{t}^{K} + v_{j}^{L} \cdot x_{t}^{L}\right) \qquad \text{for } i \in I_{Ex} \text{ and } j \in J_{EX} \quad \text{Eq 6}$$

$$\boldsymbol{LB}^{Media} \leq \boldsymbol{v}^{Media} \leq \boldsymbol{UB}^{Media} \quad \text{Eq 7}$$

$$v_{DAPDC}^{K}, v_{IPPS}^{L} = 0 \quad \text{Eq 8}$$

$$v_{leu(e)}^{Media}, v_{lys(e)}^{Media} = 0 \quad \text{Eq 9}$$

Here $\boldsymbol{S}$ is the stoichiometric matrix reported in the iAF1260 model and contains all metabolic, transport and exchange reactions. Steady-state mass balance constraints are imposed for each strain (Eq 2 and 3). The lower bounds (***LB***) and upper bounds (***UB***) are used to constrain the flux distributions (Eq 4 and 5) and concentration rate changes (Eq 7). For metabolic and transport reactions the upper limits on fluxes were set to 1000 mmol/gDW/h, and the lower limits were set to 0 or -1000 mmol/gDW/h for irreversible and reversible reactions, respectively. The upper and lower limits for the ATPM (ATP maintenance) reaction were set to 8.39 mmol/gDW/h. The upper and lower bounds for the set of exchange fluxes (set J_{EX}) and concentration rate changes used in the simulations are shown in the table at the end of this document.

The media concentration rate change ($\boldsymbol{v}^{Media}$) was calculated using Eq 6 from the exchange fluxes for each strain and the cell concentration of strain at time step t (x_t^K or x_t^L with units of gDW/L). Here J_{EX} is the set of exchange fluxes and I_{Ex} is the set of extracellular metabolites. Fluxes through reactions associated with LeuA in strain L and LysA in strain K were set to zero to reflect deletions in these two strains (Eq 8). The concentration rate changes for lysine and leucine were set to zero to ensure all the lysine produced by strain L is consumed by strain K, and leucine produced by stain K is consumed by strain L (Eq 9).

The objective function (Eq 1) in the FBA problem was first set to maximize the sum of the fluxes through the two biomass equations (by setting $c^K_{Biomass}$ and $c^L_{Biomass}$ equal to 1 and all other c values equal to 0). The biomass fluxes ($\boldsymbol{v}^K_{Biomass}$ and $\boldsymbol{v}^L_{Biomass}$) were then fixed to their optimal values and then the objective function was changed to minimize glucose uptake rates (by maximizing flux through glucose exchange reactions) needed to achieve these growth rates (by setting $c^K_{EX\_glc\_e}$ and $c^L_{EX\_glc\_e}$ equal to 1 and all other c values equal to 0).

These two optimizations were done for each time step (t) and the results used to calculate the glucose ($C_{t+1}^{glucose}$) and cell (x^K_{t+1} and x^L_{t+1}) concentrations in the next time step

(Eq 10-12) of the dFBA problem. This process was repeated until the total cell concentration reached 0.083 gDW/L (corresponding to an OD600 value of 0.2). At this point the K:L ratio and average biomass flux (i.e. growth rate) was calculated and used in **Figures 6** and **S9**.

$$x_{t+1}^{K} = x_{t}^{K} \cdot e^{(v_{Biomass}^{K} \cdot \Delta t)} \quad \text{Eq 10}$$

$$x_{t+1}^{L} = x_{t}^{L} \cdot e^{(v_{Biomass}^{L} \cdot \Delta t)} \quad \text{Eq 11}$$

$$C_{t+1}^{Glucose} = C_{t}^{Glucose} - \frac{v_{EX\_glc(e)}^{K}}{v_{Biomass}^{K}}(x_{t}^{K} - x_{t+1}^{K}) - \frac{v_{EX\_glc(e)}^{L}}{v_{Biomass}^{L}}(x_{t}^{L} - x_{t+1}^{L}) \quad \text{Eq 12}$$

The ratio of the FBA predicted growth rates ($\boldsymbol{v}^{K}_{Biomass}$ and $\boldsymbol{v}^{L}_{Biomass}$) and glucose uptake rates (-$\boldsymbol{v}^{K}_{EX\_glc(e)}$ and -$\boldsymbol{v}^{L}_{EX\_glc(e)}$) is the the biomass yield and is used in Eq 12. The starting concentrations of glucose ($C_0^{Glucose}$) and each cell type (x_0^K and x_0^L) were set to 11.96 mmol/L and 0.0026975 gDW/L). The time step used (Δt) was 0.1 hours.

TABLE: Upper and Lower Bounds for Fluxes (LB^K, UB^K, LB^L, and UB^L with units of mmol/gDW/h) and Concentration Rate Changes (LB^{Media} and UB^{Media} with units of mmol/L/hour)

**Metabolite**	**LB^K**	**UB^K**	**LB^L**	**UB^L**	**LB^{Media}**	**UB^{Media}**
Glucose	-10	1000	-10	1000	-20	1000
Oxygen	-15	1000	-15	1000	-30	1000
Ions (ca2,cl,cobalt2,cu2,fe2, k,mg2,mn2,mobd,na1,tungs,zn2)	-1000	1000	-1000	1000	-2000	1000
fe3	-1000	0	-1000	0	-2000	0
Other (nh4,pi,so4,h,h2o,cbl1)	-1000	1000	-1000	1000	-2000	1000
Leucine	value[a]	value[a]	value[b]	value[b]	0	0
Lysine	value[a]	value[a]	value[b]	value[b]	0	0
All other external metabolites	0	1000	0	1000	0	1000

[a] for uptake rate simulations the LB and UB for lysine were set to negative the specified value and for leucine were set to 0 and 1000, respectively; for release rate simulations the LB and UB for leucine were set to positive the specified value and for lysine were set to -1000 and 1000, respectively.

[b] for uptake rate simulations the LB and UB for leucine were set to negative the specified value and for lysine were set to 0 and 1000, respectively; for release rate simulations the LB and UB for lysine were set to positive the specified value and for leucine were set to -1000 and 1000, respectively.

Propagation of uncertainty with the standard curve

Quantitative PCR requires using a standard curve (Eq 13) generated from samples with a known DNA concentration. For the standard curve, the log10 transformed DNA concentration is the independent variable (denoted as x), while the measured quantification cycle (C_q) is the dependent variable (y). Parameters m and b are determined from a linear least squares estimate. The standard deviations for y and m are denoted as s_y and s_m, respectively.

For a sample of unknown DNA concentration, its log 10 transformed concentration (x) can be estimated using the standard curve and the unknown sample's C_q number. The uncertainty in x (s_x) is calculated using Eq 15 and 16, where *n* is the number of data points used to generate the standard curve and *k* is the number of measurements for the unknown sample. The standard deviation for the unknown non-transformed DNA concentration (s_z) is calculated from s_x using Eq 17.

$$y = m \cdot x + b \qquad \text{Eq 13}$$

$$s_m = \sqrt{\frac{\sum(y-b-m\cdot x)^2}{n-2}} \cdot \frac{1}{\sqrt{\sum(x_i-x)^2}} \quad \text{Eq 14}$$

$$D = \frac{s_y^2}{s_m^2} \cdot n \quad \text{Eq 15}$$

$$s_x = \frac{s_y}{|m|}\sqrt{\frac{1}{k} + \frac{x^2 n}{D} + \frac{\sum(x_i^2)}{D} - \frac{2x\sqrt{x_i}}{D}} \quad \text{Eq 16}$$

$$s_z = s_x \cdot 10^x \cdot \ln 10 \quad \text{Eq 17}$$
